# When You Do Not Get the Whole Picture: Scene Perception After Occipital Cortex Lesions

**DOI:** 10.3389/fnins.2021.716273

**Published:** 2021-12-13

**Authors:** Anna C. Geuzebroek, Karlijn Woutersen, Albert V. van den Berg

**Affiliations:** ^1^Donders Institute for Brain, Cognition and Behavior, Center for Cognitive Neuroscience, Radboud University, Nijmegen, Netherlands; ^2^School of Electrical and Electronic Engineering, University College Dublin, Dublin, Ireland; ^3^Donders Institute for Brain, Cognition and Behavior, Center for Cognitive Neuroscience, Radboud University Medical Center (RadboudUMC), Nijmegen, Netherlands

**Keywords:** occipital cortex and post-chiasmatic lesion, visual field defects, perimetry, scene perception, spatial envelope model, computational method

## Abstract

**Background:** Occipital cortex lesions (OCLs) typically result in visual field defects (VFDs) contralateral to the damage. VFDs are usually mapped with perimetry involving the detection of point targets. This, however, ignores the important role of integration of visual information across locations in many tasks of everyday life. Here, we ask whether standard perimetry can fully characterize the consequences of OCLs. We compare performance on a rapid scene discrimination task of OCL participants and healthy observers with simulated VFDs. While the healthy observers will only suffer the loss of part of the visual scene, the damage in the OCL participants may further compromise global visual processing.

**Methods:** VFDs were mapped with Humphrey perimetry, and participants performed two rapid scene discrimination tasks. In healthy participants, the VFDs were simulated with hemi- and quadrant occlusions. Additionally, the GIST model, a computational model of scene recognition, was used to make individual predictions based on the VFDs.

**Results:** The GIST model was able to predict the performance of controls regarding the effects of the local occlusion. Using the individual predictions of the GIST model, we can determine that the variability between the OCL participants is much larger than the *extent* of the VFD could account for. The OCL participants can further be categorized as performing worse, the same, or better as their VFD would predict.

**Conclusions:** While in healthy observers the extent of the simulated occlusion accounts for their performance loss, the OCL participants’ performance is not fully determined by the *extent* or *shape* of their VFD as measured with Humphrey perimetry. While some OCL participants are indeed only limited by the local occlusion of the scene, for others, the lesions compromised the visual network in a more global and disruptive way. Yet one outperformed a healthy observer, suggesting a possible adaptation to the VFD. Preliminary analysis of neuroimaging data suggests that damage to the lateral geniculate nucleus and corpus callosum might be associated with the larger disruption of rapid scene discrimination. We believe our approach offers a useful behavioral tool for investigating why similar VFDs can produce widely differing limitations in everyday life.

## Introduction

Occipital cortex lesions (OCLs) and/or post-chiasmatic lesions commonly result in the loss of visual sensitivity and blindness contralateral to the damage: a visual field defect (VFD; Zihl, 1994, 2000; Truelsen et al., 2006; Zhang et al., 2006; Celebisoy et al., 2011). These lesions can have severe debilitating consequences, disrupting people’s activities of daily living (ADLs) such as reading, driving, and their work. The difficulties are usually attributed to the *size*, *location*, and *extent* of the VFD. For example, driving is prohibited for people with OCL when they do not meet the minimum horizontal visual field extent measured with traditional perimetry techniques (Bowers, 2016). Previous research, however, shows that the VFD cannot fully explain a person’s daily-life limitations (Mueller et al., 2003; Papageorgiou et al., 2007; Gall et al., 2009). This discrepancy indicates that the current clinical practice, to judge ADL limitation in OCL patients primarily based on VFD, may be improved.

Clinically, the VFD is mapped with perimetry based on low-level visual tasks, such as the sensitivity to a local light source (static or moving) in different locations (Goodwin, 2014). Normally, our visual system needs to process complex interactions between visual signals from possibly widely separated locations in our cluttered and ever-changing visual environment (Barlow, 1961; Atick and Redlich, 1992; Van Essen et al., 1992; Field, 1994). This goes beyond what standard perimetry measures. Indeed, previous work indicated that OCL causes more global visual information processing deficits affecting gestalt recognition (Schadow et al., 2009), visual decision-making (Geuzebroek and van den Berg, 2017), useful field of view (UFOV; Woutersen et al., 2020), visual search (Machner et al., 2009), and recognition and spatial navigation (Peyrin et al., 2005; Cavézian et al., 2010, 2015; Perez et al., 2013). More specifically, left VFD significantly disrupts scene categorization tasks, which even suggests a hemispheric specialization (Fink et al., 1997; Chokron et al., 2000; Hana et al., 2002; Lidaka et al., 2004; Peyrin et al., 2005; Cavézian et al., 2010, 2015; Musel et al., 2013; Perez et al., 2013; for a review, see Kauffmann et al., 2014). In this study, we explore a more naturalistic approach based on scene perception to better characterize the consequences of OCL.

Visually identifying one’s environment and its opportunities is an important competence (Kaplan, 1992; Schyns and Oliva, 1994; Thorpe et al., 1996; Oliva and Torralba, 2001; Joubert et al., 2007; Greene and Oliva, 2009, 2010). It allows us to choose the appropriate course of action rapidly (19–67 ms), almost in a reflex-like manner (Kaplan, 1992; Thorpe et al., 1996). This global understanding, or the so-called gist of the scene, is very robust to reduced image resolution, or restricting visibility to the far periphery (Rousselet et al., 2005; Larson and Loschky, 2009; Wolfe et al., 2011; Boucart et al., 2013; Geuzebroek and van den Berg, 2018). Furthermore, it seems to precede the processing of finer details (Schyns and Oliva, 1994; Torralba and Oliva, 2003; Guyader et al., 2004; Hegdé, 2008; Musel et al., 2012, 2013; Trouilloud et al., 2020). These observations led many to believe that the gist of a scene is the result of the rapid pooling and summarizing of visual information over large visual areas within a highly parallel, feed-forward network (Thorpe et al., 1996; Rousselet et al., 2002). Scene processing thus seems to rest upon an important component of non-local visual information integration. Hence, extending perimetry with characterization of scene processing abilities in people with OCLs may provide an avenue to better understand ADL limitations. Unilateral V1 and post-chiasmatic damage may affect scene perception in a global manner, causing performance loss beyond that expected from a local occlusion by the VFD.

To examine the effects of V1 or post-chiasmatic lesions in detail, we seek a method to unambiguously compare OCL participants’ performance on a scene discrimination task with that of healthy observers. Simply matching healthy observers on age, gender, socioeconomic status, and so on would not take into account the wide variation of patients’ defects. We explore the potential of simulating their lesions in the spatial envelope model (e.g., GIST model; Oliva and Torralba, 2001), a computational model that summarizes the scene by coarsely filtering the spatial frequency distributions using a global Fourier transformation. Even though there are no known anatomically plausible mechanisms that could apply such a transformation on the image, the model is highly successful in predicting human scene perception (Oliva and Torralba, 2001; Greene and Oliva, 2009; Ehinger and Rosenholtz, 2016).

Here, we compare performance of OCL participants and healthy observers, in whom a field defect was *simulated by a mask*, on a rapid scene discrimination task, comparing both groups with the prediction made by the GIST model simulations. We expect that the healthy observers will only suffer from the information loss due to the occlusion of part of the visual scene, while the performances of the OCL participants may be affected differently as the lesion, or adaptation to it, could affect the processing capacity of the visual network itself.

## Materials and Methods

### Participants

Seventeen adult participants (OCL participants; mean age 58 ± 18.9 years; three females and 14 males) with a VFD following OCLs or post-chiasmatic lesions were recruited and invited to the Donders Institute for Cognitive Neuroscience in Nijmegen. In 13 OCL participants, the VFD was a consequence of ischemic or hemorrhagic stroke; in three other, the VFD was a side effect of surgical tumor removal; and in one OCL participant, the VFD resulted from the surgical removal of a knot of blood vessels (Table 1 and Supplementary Table 2). Additionally, 18 adult participants (age 57 ± 15 years; 11 females and seven males) were recruited as healthy observers in the control group. All participants had normal or corrected-to-normal visual acuity as measured with the Freiburg test (Bach, 1996, 2007). This study was part of a larger project, which the local ethics committee of Radboud University Medical Center in accordance with the Declaration of Helsinki approved all experiments (2016–2635); and each participant gave written informed consent prior to testing.

**TABLE 1:** Demographic and clinical details of the OCL participants and the averages of the healthy controls.

OCL no.	Age (years)	Months after lesion	Lesion etiology	VFD location	BFI	Line bisection (mm)	Visual acuity (logMar)	iUFOV (ms)^+^	Total VFQ scores^+^
P01	68	8	Ischemic	L	−0.39	0.2	−0.13	−	90
P02	26	295	Hemorrhagic	R	−0.25	0.9	−0.20	63	86.9
P03	52	51	Surgery (tumor)	L	−0.51	3.2	0.20	918	61.3
P04	71	20	Ischemic	L	−0.42	−0.9	0.08	372	63.9
P05**	22	19	Surgery (tumor)	L	−0.58	2.2	−0.09	552	61.3
P06	63	20	Ischemic	R	−0.32	3.4	0.19	118	41
P07	25	39	Surgery (cavernoma)	R	−0.44	4.6	−0.18	168	76.2
P08	70	30	Ischemic	L	−0.42	3.9	−0.10	−	97.1
P09	75	31	Ischemic	L	−0.20	0.9	0.23	278	78.3
P10	74	35	Ischemic	R	−0.17	0.1	0.23	278	77.5
P11	64	32	Ischemic	R	−0.33	2.9	−0.16	208	87.3
P12	60	30	Hypoxia	L	−0.43	7.5*	−0.12	53	80.3
P13	36	84	Ischemic	L	−0.42	−2.0	−0.13	178	63.5
P14	50	151	Ischemic	L	−0.36	3.0	−0.15	108	91.5
P15	81	68	Ischemic	L	−0.53	−4.0	0.44	−	59.6
P16	70	33	Hemorrhagic	L	−0.42	4.8	0.09	828	40.9
P17	48	161	Surgery (tumor)	L	−0.48	−0.5	−0.10	−	76.8
OCL (SEM)	56.2 (4.6)	58.6 (17.6)	–	–	–	4.9 (2.5)	0.00 (0.04)	370 (78)	72.6 (4)
Control (SEM)	56.9 (3.5)	–	–	–	–	–	−0.10 (0.07)	142 (16)	94.1 (1)
Stats	*t*(33) = 0.13, *p* = 0.89						*t*(33) = −1.2, *p* = 0.25	*t*(28) = −2.5, *p* = 0.019*	*t*(33) = −5.8, *p* < 0.001*

*Line bisection is the deviation relative to VFD (positive values represent a deviation toward the defect).*

*Blind Field Index (BFI) reflecting the average blind field size as a fraction of the whole field.*

*Visual acuity (VA) is measured with the Freiburg test (Bach, 1996, 2007).*

*A logMar higher than 0.1 would be marked as below average.*

*OCL, occipital cortex lesion; VFD, visual field defect; iUFOV, useful field of view test in the intact field; VFQ, Visual Function Questionnaire; SEM, standard error of the mean.*

*^+^For full description of the iUFOV test and its correlation with VFQ, see Woutersen et al. (2020).*

*p < 0.05.

**Humphrey low test reliability (see Supplementary Table 1).

### Procedure

**FIGURE 1:**
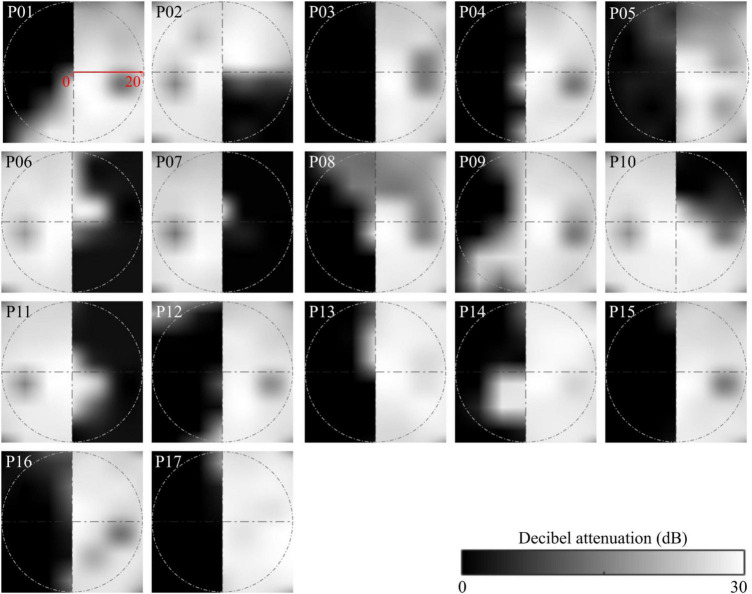
Visual field maps of the OCL participants. We used central 30-2 threshold Humphrey perimetry. Maps show averaged sensitivity of left and right eyes. OCL participants’ VFDs are, as a rule, limited to one hemifield, due to the damage caused by contralateral post-chiasmatic or V1 lesions. Each panel shows the measured VFD of one participant interpolated on a 256 × 256 pixel grid representing a visual extent of 20° radius corresponding to the dimensions of our scene images (see P01). Luminance sensitivity is given by decibels of attenuation of the target, shown in the grayscale. No attenuation of light intensity is marked as 0 dB, which indicates that the participant was not able to detect the stimulus at the highest luminance.

Prior to their visit, participants were asked to fill out a Dutch version of the National Eye Institute—Visual Function Questionnaire-25 (NEI VFQ-25), a vision-related quality of life questionnaire (Mangione et al., 2001; see for detailed description Woutersen et al., 2020). Then, the Freiburg visual acuity test was performed to measure visual acuity (Bach, 2007, 1996). Additionally, OCL participants performed a line bisection task to look for signs of neglect (Table 1) and a central 30-2 threshold Humphrey^®^ to map the VFD extent and shape and extract the visual field index (VFI) (Carl Zeiss Meditec Group, Jena, Germany; see Figure 1 and Supplementary Table 1 for a summary of the reliability indices). The VFI is an indicator for the total amount of field loss ranging from 1 (normal) to 0 (blind). A Blind Field Index (BFI) was calculated by subtracting the VFI from the maximum performance (e.g., 0 is no blind field, and −1 is completely blind) averaged cross both eyes. The researchers (AG and KW) online monitored the participants’ attention to ensure the quality of the Humphrey^®^ measurement, and the test reliability indices in Supplementary Table 1 were used to evaluate whether the measurement could be used. Last, participants would perform two psychophysical tests: the scene discrimination task as described in this paper and the UFOV test in the intact field (iUFOV), described in Woutersen et al. (2020).

Before the main experiment, in each participant, the scene presentation durations required to achieve maximum performance for a noise-free scene in either discrimination task were determined. Each staircase comprised 50 trails (as described section “Scene Discrimination Task”) during which the image presentation time in milliseconds converged to a threshold of approximately 80% correct. The resulting presentation times were multiplied by 1.5 to promote best performance throughout the rest of the study. The refresh rate was 60 Hz, allowing the shortest presentation time of 16.7 ms; the maximum image presentation time was set to 300 ms.

### Experimental Setup

The scene discrimination task was performed on a 3,840 × 2,160 (screen) pixel iMac Retina 5K display (Apple, Cupertino, CA, United States). The refresh rate was 60 Hz, allowing the shortest presentation time of 16.7 ms. Stimuli were generated with custom-made software in Matlab^®^ R2016a (MathWorks^®^, Inc., Natick, MA, United States) using the Psychtoolbox-3 (Brainard, 1997; Pelli, 1997; Kleiner et al., 2007). A participant’s head was stabilized using a head and chin rest. Eye movements and blinks were monitored using an EyeLink 1000 (SR Research, Kanata, ON, Canada) to ensure fixation through the experiment. A standard 9-point calibration with validation procedure was performed before the psychophysical task and was repeated when the eye tracking was lost.

### Scene Discrimination Task

**FIGURE 2:**
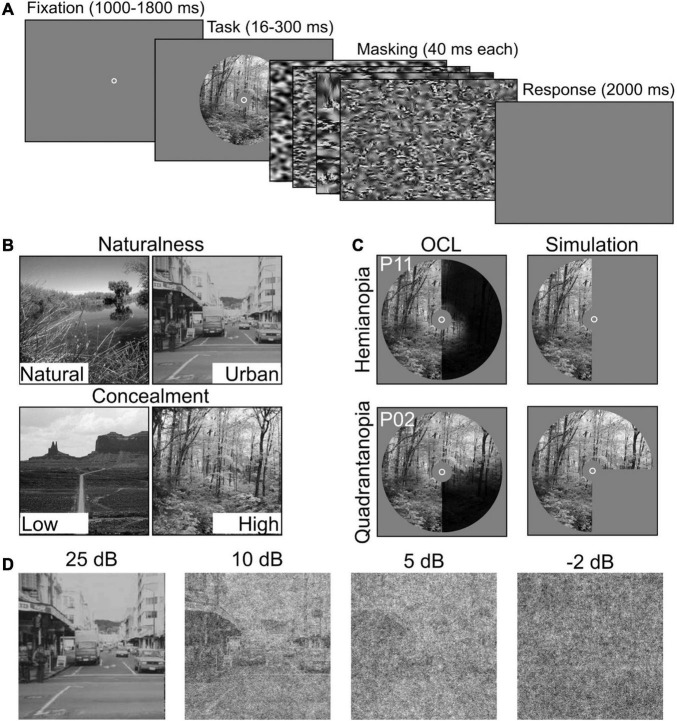
Experimental setup and stimulus conditions. **(A)** Participants performed two scene discrimination tasks. Each started with a random fixation interval (between 1,000 and 1,800 ms), followed by scene images. Scene presentation time was individually determined. The scene images were backward-masked with four noise images presented for ms each. After the masking interval, participants were asked to categorize the scene as accurately and quickly as possible. **(B)** Example scenes for the two scene discrimination tasks (Naturalness and Concealment) and their two levels. **(C)** Field defects of two OCL participants (P2 and P11) compared with simulated VFD occlusion. **(D)** Examples of the systematic degradation using four levels of SNR, i.e., 25, 10, 5, and −2 dB.

Participants performed two scene discrimination tasks: a naturalness discrimination task (e.g., natural vs. urban scenes) and a concealment discrimination task (e.g., high vs. low concealment scenes). Per discrimination task, 100 grayscale scene photographs (256 × 256 image pixels—larger than screen pixels) were used that were previously classified as stereotypical for each of the categories (Greene and Oliva, 2009). A description of the categories can be found in Table 2, and examples are in Figure 2B. All images were gamma corrected and equalized for mean luminance and contrast using the SHINE toolbox (Willenbockel et al., 2010).

**TABLE 2:** Descriptor of the categorization tasks and the instructions as presented to participants.

Category	Descriptor	Task
Naturalness	Natural	The scene is a natural environment.
	Urban	The scene is an urban environment.
Concealment	Low	You would be easily seen while standing in the scene, and there are not many places to hide objects.
	High	The scene contains many accessible hiding places, and there may be hidden objects.

Each trial started with a fixation circle at the center of the screen. After a random fixation interval (between 1,000 and 1,800 ms), a scene image was shown in a circular aperture of 20^*circ*^ (radius), with individualized presentation duration described in section “Procedure”. The scene image was followed by backward-masking sequence, consisting of four noise images each presented for 50 ms. The noise images were generated with a texture-synthesis algorithm (Bacon-Mace et al., 2005; Portilla and Simoncelli, 2007; Greene and Oliva, 2009) that preserves the first and higher-order statistics of the scene image for optimal masking (Loschky et al., 2010). When the noise images disappeared, participants were asked to indicate the appropriate category as accurately and as fast as possible (Figure 2A). Importantly, participants were instructed to maintain central fixation throughout the experiment. Attempts to reduce the effect of small eye movements were made by occluding the central 5° diameter of the scene (Hegdé, 2008) and by giving verbal feedback based on the eye tracker. Trials in which participants failed to maintain fixation were discarded.

Scene discrimination tasks (naturalness or concealment) were block-randomized, and signal-to-noise ratio (SNR) was randomized within the blocks. All participants, OCL participants and controls alike, were subjected to a whole field condition (indicated as noVFD in controls). The simulated hemianopia occlusion (hVFD) defect locations (left vs. right) were counterbalanced between controls (*N* = 18) and thus “between-parameters.” Controls were asked to return on a different day to perform both discrimination tasks with an additional quadrant simulated occlusion; i.e., the left-up qVFD, left-down qVFD, right-up qVFD, and right-down qVFD were counterbalanced between controls (*N* = 16).

Scene information was degraded with pink noise (1/f) at four different SNR levels (SNR conditions), i.e., −2, 5, 10, and 25 dB (see Figure 2D). This was done because previous studies suggest that OCL participants are more sensitive to the disruption by noise (Bender and Teuber, 1946; Rizzo and Robin, 1996; Paramei and Sabel, 2008; Paramei et al., 2017). The different types of VFDs were simulated in healthy controls, by masks occluding specific parts of the scene image (Figure 2C). Performance was assessed in a “no visual defect” condition (noVFD), for a left or right hemianopia simulation (lVFD/rVFD), and for a quadrantanopia simulation (left-up VFD, left-down VFD, right-up VFD, and right-down VFD, summarized as qVFD).

### Categorization by GIST Model

Our aim was to compare the performances of OCL participants with healthy controls, by using an objective reference provided by a successful model of scene perception (GIST, Oliva and Torralba, 2001) and its performance when part of the scene is not visible. A support vector machine (SVM) was trained using GIST features to build two classifiers—one for naturalness and one for concealment—to compute the best hyperplane to distinguish between the two scene categories (e.g., natural vs. urban and low vs. high concealment) using noise-free image (see below for description of the training database).

The GIST model extracts scene information from a multiscale representation of the retinal image. The image is characterized by the power distribution across several orientations and spatial frequencies. This can be interpreted as the averaged output of local receptive fields with different orientation and spatial frequency preferences into a global receptive field. GIST features are computed by multiplying each Fourier transformed image with 48 Gabor filters, using eight orientations and six spatial frequencies using the open source Matlab code provided by Oliva and Torralba (2001)^1^. These were then transformed back into image space, resulting in 48 Gabor-filtered images for each scene. These filtered images were subdivided in 4 × 4 grid equal-sized regions and averaged across all pixels per grid point. This resulted in 768 image features per scene image that were used as input to the SVM. Lesions were applied by simply occluding part of the input image corresponding to the simulated lesions for controls (see Figure 2C) or the measured VFDs for OCL participants (see Figures 1, 2C). The fact that this model uses the spatial frequency domain makes it inherently global; the local masking will thus have global effects, as they influence the power distribution across spatial frequencies.

#### Training of the Model

To train the SVM, a large and diverse dataset of 10,000 images was first created and validated per scene discrimination task (naturalness and concealment). With the use of the SUN database and its scene hierarchy (Xiao et al., 2016), 5,000 images was randomly selected for each naturalness level (natural and urban). The natural images were further divided in low or high level of concealment. Concealment was selected based on the description of Greene and Oliva (2009); “How efficiently and completely a human would be able to hide in a space, or the probability of hidden elements in the scene that would be difficult to search for.” Scenes can range from low concealment, which refers to complete exposure in a sparse space, such as deserts, oceans, and fields, to high concealment with dense, variable surfaces and many objects, such as forests and riverbeds (Greene and Oliva, 2009). Five thousand images were again selected for each concealment level (low and high) derived in part also from Places365-Standard, as there were not enough images left in the SUN database (Zhou et al., 2018). The dataset was manually checked to exclude (a) ambiguous images (cultivated field or landscaped gardens), (b) images that could be recognized solely on the recognition of a single large object, and (c) unusual images (distortion of colors or borders, very blurry or noisy). All training images were down-sampled to 256 × 256 pixels and converted to gray values, and local contrast was normalized before the GIST features were computed.

#### Testing Computational Models

To test the model, we applied a semi-random split procedure such that the subset of scene stimuli (described in section “Scene Discrimination Task”) used for each participant in each condition (SNR condition, discrimination task, and VFD simulation) was used also to compute the model prediction of that individual’s sensitivity. Each participant received individually randomized samples from a large database of images. The semi-random split allows us to find a difference between human and GIST performance that cannot be attributed to differences in the stimulation.

### Statistical Analysis

#### Human Performance

For each condition, we calculated observed sensitivity (*d*′) and bias, which combine the hit and errors rates in a bias-free estimate of performance (Kingdom and Prins, 2010). To assess sensitivity as a function of defect extent and location in the healthy controls, we first performed a mixed-factor ANOVA with discrimination task (naturalness and concealment) × SNR levels (−2, 5, 10, and 25 dB) × VFD extent (hemianopia and quadrantanopia) as within-parameters and VFD location (left and right) as between-parameters. Furthermore, to compare the OCL participants’ performance with that of the controls, the researcher first crudely categorizes the OCL participants’ as either (1) a left or a right VFD or (2) a quadrantanopia or a hemianopia. To compare the four OCL participants groups with the healthy controls with the simulated VFD, we applied a separate mixed-factor ANOVA for each group, e.g., left quadrantanopia (*N* = 9), right quadrantanopia (*N* = 3), left hemianopia (*N* = 3), or right hemianopia (*N* = 2). We used discrimination task (naturalness vs. Concealment) × SNR levels (−2, 5, 10 and 25 dB) as within-participants parameters and group (OCL participants category vs. Controls) as the between-participants parameters. To correct for multiple comparisons, we applied the false discovery rate (FDR) correction to the *p*-values.

#### Model Performance

Accuracy of the GIST classifier is notoriously more susceptible to noise (Serre et al., 2007; Geirhos et al., 2017; Tadros et al., 2019) than human observers, making it difficult to interpret the effects of SNR degradation for such a measure. We observed that for SNR levels < 10 dB, the classifier has a strong bias toward one level in the discrimination tasks—Natural and High concealment specifically—yet sensitivities of the two levels are still distinguishable. By assuming that the GIST classifier output is normally distributed also for lower SNR, we can still compute the estimated sensitivity (d^′) for the GIST classifier.

Whether the GIST classifier’s performance is significantly better or worse than the healthy controls’ is calculated from the sensitivity difference r^=d^′−d′ between the estimated sensitivity by GIST (d^′), and the observed sensitivity (*d*′) in the healthy controls (Tadros et al., 2019). Average values of $\hat{r}$ significantly greater than 0 correspond with the GIST classifier being more sensitive than human classification and vice versa. We therefore used a Bayesian *t*-test to test if the average sensitivity difference ($\hat{r}$) for healthy controls was significantly different (Kass and Raftery, 1995; Rouder et al., 2012; Morey and Wagenmakers, 2014; Schoenbrodt and Wagenmakers, 2017). This *t*-test derives a Bayes factor (BF) by comparing the fit of the data for the null hypothesis (equal sensitivity: $\hat{r}$ = 0) with the alternative hypothesis using Bayesian information criteria. The BF gives an estimation of the likelihood that the null hypothesis is true (Jeffreys, 1961; Lee et al., 2013). For example, if we accept the null hypothesis, we can use the Bayes factor to estimate of how likely the null hypothesis was compared with the alternative. A Bayes factor of 10 suggests that the null hypothesis is 10 times more likely. FDR correction was applied to account for multiple comparisons. We used the natural logarithm of the Bayes factor > 4.6 as a cutoff for strong evidences.

#### Occipital Cortex Lesion Participants

To evaluate whether an OCL participant’s performance is different from what the extent and location of the VFD would predict, OCL participants with a BFI < −0.375 are crudely classified as left quadrantanopia (P09, P14; *N* = 2) or right quadrantanopia (P02, P06, P10, and P11; *N* = 4), and left hemianopia (P03, P04, P05, P08, P12, P13, P15, P16, and P17; *N* = 9) or right hemianopia (P07; *N* = 1). As described in the previous paragraph, we calculated the sensitivity difference between the OCL participant and the predicted sensitivity by the GIST classifier using the participant’s VFD. Average score significantly lower than 0 indicates that the OCL participant performs worse than can be explained by occlusion (as in healthy observers with a simulated VFD), and vice versa.

Last, we used Pearson’s correlation to analyze the sensitivity difference between OCL participant and the predicted sensitivity by the GIST classifier with both the VFQ-25 and iUFOV scores to see how difficulties in scene perception translate to real-life impairments. As this is an exploratory addition to this study, we did not correct for multiple comparisons to retain sensitivity.

### Structural Scans

Existing structural scans (CT or MRI scans) of the OCL participants were collected *post hoc* to explore possible information of the extent and location of the lesion. Unfortunately, we were only able to acquire a heterogeneous and incomplete imaging dataset (data of only 12 OCL participants were available), admittedly allowing only weak conclusions. These scans were brain extracted, aligned to Montreal Neurological Institute (MNI; Montreal, Quebec, Canada) standard space and automatically segmented in FMRIB Software Library v6.0 (FSL, Smith et al., 2004). The lesions were manually drawn in; using this segmentation and the Jülich histological probabilistic map gave us an indication which areas were affected by these lesions (Eickhoff et al., 2005).

## Results

To evaluate our procedure to optimize presentation time for each participant, we applied a mixed ANOVA to the average thresholded presentation times as a function of group and discrimination task. We observed no effect of group [*F*(2, 22) = 2.25, *p* = 0.13] and a significant difference between discrimination tasks [F(1, 22) = 16.0, *p* < 0.001]. Both groups, healthy controls and OCL participants alike, needed longer presentation duration in the concealment task [μ = 0.22 s (0.16, 0.27)] than in the naturalness task [μ = 0.12 s (0.07, 0.17)].

The individually determined presentation durations were further used throughout the experiment. To further determine if our tailoring of presentation time duration resulted in comparable performance across tasks, we applied a mixed-factor ANOVA to sensitivity as a function of SNR (−2, 5, 10, or 25), simulated VFD extent (noVFD, qVFD, or HVFD simulations), and discrimination task (naturalness vs. concealment) as within-parameters and simulated VFD location (left vs. right) as between-parameters. Mauchly’s test of sphericity indicated that the assumption of sphericity had been violated, and the Greenhouse–Geisser corrected *p*-values were therefore reported. There was no significant effect of discrimination task [*F*(1, 10) = 0.10, *p* = 0.8]. There were also no significant effects of VFD location (left vs. right) on controls’ sensitivity [*F*(1, 10) = 0.41, *p* = 0.5]; occluding the left part or the right part of the visual field on average did not change performance. We pooled the sensitivities of the controls across the two discrimination tasks and the VFD location, for plotting purposes only.

Next, we address the following issues: (1) what are the effects of local occlusions on scene discrimination sensitivity in healthy controls, and can the GIST model describe these effects correctly? (2) what is the group effect of occipital lesions in patients compared with the occlusion effect in healthy controls? and (3) how does each patient’s performance loss due to the occipital lesion compare with the performance loss due to that individual’s occlusion effect as estimated by the GIST model?

### Behavioral Performance of Healthy Controls

#### Effects of Local Occlusions in Healthy Controls

**FIGURE 3:**
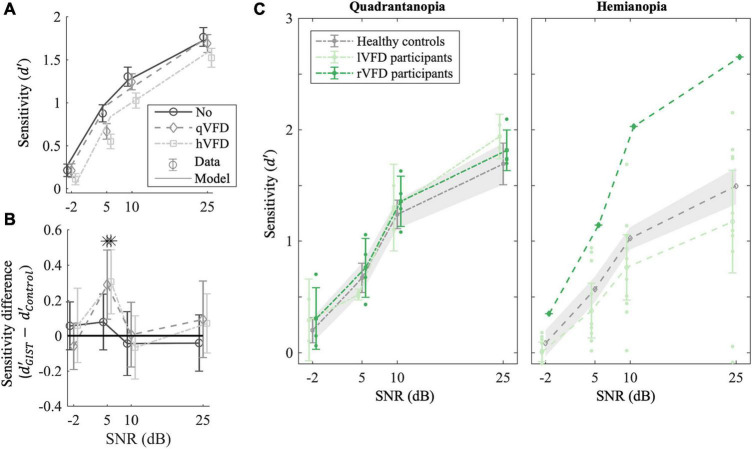
Effect of local occlusions on participants’ performance in scene discrimination tasks. **(A)** Average empirical (markers) and simulated sensitivity (lines) as a function of signal-to-noise ratio (SNR) and the extent of the simulated VFDs (qVFDs or hVFD) for healthy observers. **(B)** GIST classification accuracy relative to healthy controls’ performance. The sensitivity difference of the classifier to human behavior is plotted as a function of the simulated VFD and SNR. **(C)** Average sensitivity for participants with quadrantanopia VFD (P02, P06, P09, P10, P11, and P14) and (right panel) participants with hemianopia VFD; each group was compared with corresponding simulated VFDs in healthy controls. Additionally, the sensitivity per SNR condition of the individual participant with occipital lesion is plotted, illustrating the large variability in the OCL group (see also Figure 4). Error bars and shaded areas show the CI_95%_. *p < 0.05 after false discovery rate (FDR) correction.

We applied a mixed-factor ANOVA to sensitivity and bias as a function of SNR, simulated VFD extent and discrimination task as within-parameters, and simulated VFD side as between-parameters. Figure 3A shows that healthy controls’ *sensitivity* was significantly affected by SNR [*F*(3, 28) = 113.99, *p* < 0.001]. Not surprisingly, participants become increasingly less sensitive as the SNR decreases (−2 < 5 < 10 < 25 dB, *p*’s < 0.001). There was a significant interaction effect on healthy control’s *bias* between SNR and discrimination task [*F*(3, 32) = 14.46, *p* < 0.001]. Participants gradually move their bias in the naturalness task from urban (25 dB) toward natural when noise increases (−2 > 5 > 10, *p*’s < 0.001), while their bias in the concealment task moves toward high concealment when noise increases (−2 < 5 < 10 < 25 dB, *p*’s < 0.05). Furthermore, in Figure 3A, healthy controls’ sensitivity appears significantly affected by the VFD extent [*F*(2, 32) = 8.85, *p* = 0.002]. Healthy controls become less sensitive for a simulated hVFD than for noVFD [Δ*d*′ = −0.22 (−0.36 −0.09), *p* = 0.002] and identically so for simulated qVFD [Δ*d*′ = −0.12 (−0.25 < 0.001), *p* = 0.04], although barely.

These results characterize the effect of occlusion of part of the scene on discrimination performance by observers with a healthy visual network. Next, we investigated whether the GIST model captures those characteristics appropriately.

#### Simulated Lesions in the GIST Model

To verify the use of the GIST model, we computed the average sensitivity difference ($\hat{r}$) of the sensitivity estimated by GIST classifier and the observed sensitivity in the healthy controls as plotted in Figure 3B. If the sensitivity difference is greater than 0, it would mean that the GIST classifier is more sensitive than human classification and vice versa. The *t*-test after FDR correction shows that SNR levels of 5 dB are significantly different from 0 for the two VFD simulations [*t*(30) = 3, *p* = 0.02, BF = 20 and *t*(28) = 3.5, *p* = 0.03 BF = 7.8, for qVFD and hVFD, respectively]. At SNR of 5 dB, the GIST model is more sensitive in the scene discrimination task and specifically overestimates the performance when a VFD is simulated. For all other SNR levels (−2, 10, and 25 dB), the sensitivities are not significantly different with all BF > 3, indicating moderate evidence for the null hypothesis ($\hat{r}$ = 0). At the lowest SNR level (−2 dB), however, in this condition, both model and human observer reach the floor. Taken together, this suggests that there is moderate evidence that the model can meaningfully predict the sensitivity of healthy controls and the effect of VFD for the two highest SNR levels. To benchmark OCL participants’ performance, we thus limited our analysis to the two larger SNR levels (10 and 25 dB).

### Effect of Occipital Lesions?

**FIGURE 4:**
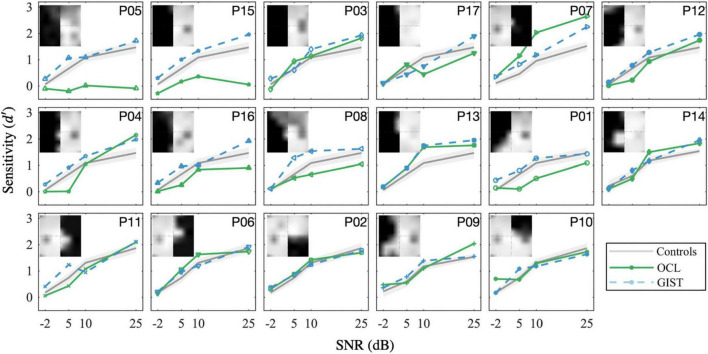
OCL participants’ observed sensitivity (**d**′) as compared with healthy controls crudely matches predicted sensitivity as well as the estimated sensitivity (d^′) by the GIST model. Each panel shows OCL participants with their VFD, sorted by the Blind Field Index from the largest (P05) to the smallest (P10). Note that the performance of the OCL participants (greens) does not always correspond to the extent of the VFD, e.g., comparing participant P15 and P03. Error bars and shaded areas show the CI_95%_.

To examine the if occipital lesions modulate performance on a scene perception task beyond what local occlusions would predict, we compare the OCL participants with the healthy participants with the simulated lesions (none, qVFD, hVFD) and the GIST model (see Figure 3C for the group averages and Figure 4 for the individual OCL analysis).

We first classified the OCL participants as being left quadrantanopia (P09, P14; *N* = 2), right quadrantanopia (P02, P06, P10, and P11; *N* = 4), left hemianopia (P03, P04, P05, P08, P12, P13, P15, P16, and P17; *N* = 9), or right hemianopia (P07; *N* = 1). Separate mixed-factor ANOVAs were applied to compare the four categories with the healthy controls with simulated field defects. This showed that there were no significant group differences of sensitivity in the quadrantanopia class [*F*(1, 10) = 0.3, *p* = 0.6 and *F*(1, 8) = 0.5, *p* = 0.5, for left qVFD and right qVFD, respectively; Figure 3C, left panel], or for the hemianopia class [*F*(1, 12) = 0.89, *p* = 0.3 and *F*(1, 10) = 6.3, *p* = 0.03, for left hVFD and right hVFD, respectively; Figure 3C, right panel].

On average, OCL participants do not significantly perform worse on the scene discrimination task than healthy observers with a field defect that is simulated by the occluding part of the scene. Clearly, the OCL for left and right defects plotted in Figure 3C and the individual OCL performances in Figure 4 show a much larger variability.

This indicates that the average sensitivity might not reliability present each individual patient. As we showed before, the *extent* of the occlusion significantly modulates performance in healthy controls. It is likely that part of that variability of OCL participants stems from the variability of their VFD *extent* and *shape* (see Figure 4). Clearly, sensitivity in many patients (green curves in individual panels of Figure 4) strongly deviates from the average performance of healthy controls (gray line and shaded area showing its CI_95%_) to the corresponding simulated field defect; however, the same goes for the VFD. Take, for example, OCL participants P04 and P13. Both have been “crudely” classified within the hVFD group; however, they both do not have a full hemianopia. When looking at the GIST model (blue line in Figure 4), we see that the comparison with the healthy controls might overestimate their “better” than expected performance.

**FIGURE 5:**
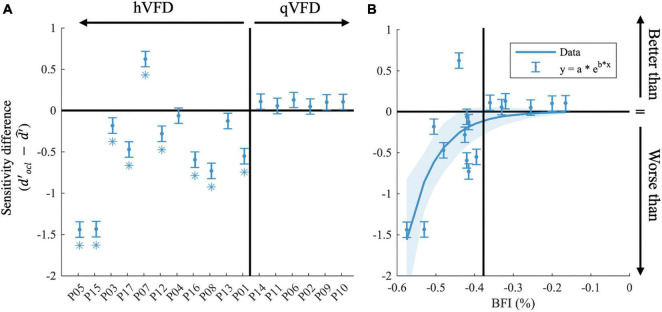
Effects of occipital lesions. **(A)** The sensitivity difference (${\text{d}}_{O\,C\,L}^{{}^{\prime }}-{\hat{\text{d}}}^{{}^{\prime }}$) comparing the observed sensitivity of the OCL participants with that of the estimated sensitivity of the GIST model, sorted by the Blind Field Index from the largest (P05) to the smallest (P10). **(B)** Sensitivity difference as a function of BFI. In both plots, the black horizontal line indicates when the observed sensitivity is not different from the predicted sensitivity loss by occlusion; the vertical black line separates the hVFD and the qVFD participants. Error bars show the CI_95%_ and *p-values < 0.05 after false discovery rate (FDR) correction.

To further investigate, we averaged across discrimination tasks and SNR levels (10 and 25 dB), the sensitivity difference between OCL participant, and the GIST model using the individual’s VFD ($\hat{d^{\prime }})$ in Figure 5A. Note that the null hypothesis, $d_{O\,C\,L}^{\prime }-\hat{d^{\prime }}=0$, tests the assumption that performance loss is solely due to the occlusion of part of the visual scene as in healthy observers. Individual *t*-tests after FDR correction shows that eight OCL participants (P01, P03, P05, P08, P12, P15, P16, and P17) perform worse than expected for their simulated VFD by the model [*p* < 0.05, with ln(BF) > 4.6, e.g., strong evidence that the H_0_ is rejected]. One OCL participant, P07, performs better than predicted [*p* < 0.05, with ln (BF) > 4.6]. These results reject the hypothesis that performance loss is solely due to the scene occlusion. Interestingly, Figure 5A shows that there is an exponential relationship between sensitivity difference and the size of the BFI [slope, e.g., *b* = −13.3 (−22 −4.8), adjusted *R*^2^(15) = 0.53]. This trend would suggest (with exception of P07) that larger BFI results in exponentially more reduction of scene discrimination performance, if the larger VFDs are found in patients with more damage to the network’s processing capacity. Furthermore, there was no significant correlation between the sensitivity difference and the VFQ-25 (*R* = 0.16, *p* = 0.53) or the iUFOV (*R* = −0.5, *p* = 0.08).

In this study, we did not collect structural scans ourselves. However, we were able to secure these data for 12 patients (see Supplementary Figure 1 and Supplementary Table 2) by contacting their caregivers at the time of the event. The limited sample precludes strong conclusions, but we do notice a trend that the location of the lesion rather than its extent has a large impact on the patient’s performance. Namely, all lesions of participants that score worse than predicted involve the corpus callosum and/or subcortical areas, i.e., the lateral geniculate nucleus (LGN), while these areas were intact in the other patients. Additionally, referring to Table 1, one can observe that only one of the OCL participants who performed worse than predicted showed slight signs of neglect, and all have a left-sided VFD.

## Discussion

We examined the extent to which post-chiasmatic and V1 lesions disrupt scene perception. If the limitation arises exclusively from an occlusion of the incoming information, an OCL participant should perform equally compared with a healthy observer when a similar VFD is simulated with a mask. To handle the large variation among OCL participants’ VFD and performance, we used the GIST model for scene perception. Occlusion of the input to this model implements the assumption that disruption of the ascending visual pathways can be equated to occluding certain parts of the scene but does not affect the capacity of the network to process the information. The GIST model successfully predicted the performance reduction by occlusion and noise in healthy observers down to an SNR of about 10. Using the GIST model, we should thus be able to predict how occipital lesions, under the above assumption of information reduction by the occlusion, would influence OCL participants’ performance in the scene discrimination task. This was true in nearly half of our OCL participants. Most of the other OCL participants performed worse than predicted, breaking the assumption that the field defect only reduces the access to the visual information while not interfering with the ability to process the global scene.

One OCL participant performing better indicated that he/she had access to more information of the scene than his/her VFD would mask. Interestingly, a recent study showed that in some OCL participants, neural responses can be evoked in parts of the occipital cortex from visual stimuli placed in the absolute VFD (Papanikolaou et al., 2014). It is tempting to suggest that such people may also be able to perform better on scene discrimination tasks than their Humphrey field defect indicates, precisely when such activity would contribute to visual processing in a non-localized way as probed by scene tasks but subthreshold for localized processing as in perimetry. Because such neural responses from the absolute field defect correlated not only with spared islands of activity in occipital but throughout visually responsive cortex, and as this indicates a better prognosis for visual field recovery by visual training (Elshout et al., 2018), OCL participants with unexpectedly good performance on scene discrimination might profit in particular from restitution therapy.

Hence, we believe that our modeling approach could be a potentially important diagnostic tool to further characterize patients’ defects as (1) only limiting access to information or as (2) limiting access plus a reduced ability to process the remaining information or (3) indicative of a neural “reserve” for visual processing.

### Hemispherical Lateralization Effects of Occipital Stroke?

Previous studies suggested that V1 damage constrained to the right hemisphere (resulting in a left VFD) affects scene discrimination more than lesions to the left hemisphere (Coubard et al., 2008; Cavézian et al., 2010, 2015; Perez et al., 2013). In these studies, people with OCL performances were directly compared with healthy observers. While such a comparison reveals the extent of the functional deficit caused by the defect, it does not differentiate between the contributions of the loss of visual input and damage to the network reducing its capacity for visual processing. Our results hint at some hemispherical lateralization effects. The cursory analysis of our data suggests that left VFD participants are less sensitive and need longer presentation times (although not significantly) than right VFD participants. Our modeling approach when accounting for the various individual VFDs shows only a moderate trend that left VFD participants are less sensitive but shows no significant effect. We also do not see a hemifield effect in our healthy observers, as there was no significant performance difference between left or right VFD simulations (see section “Effect of Occipital Lesions?”). In contrast, all OCL participants in the “worse than the GIST model group” were lVFD. However, we had very few participants with a right VFD. Hence, we feel that the extended damage into subcortical structures that was consistently present in this group provides for now at least an equally likely explanation of their deficit rather than the side of their field defect.

Our backward-masking protocol has been suggested to largely influence higher-order feedback as well as the parvocellular pathway (Breitmeyer and Ogmen, 2000; Enns and Di Lollo, 2000; Breitmeyer et al., 2006). Both these pathways could contribute to refine the initial “global and coarse” perception of the scene. To us, all these open the possibility that previously found hemispheric asymmetry might not necessarily originate in the ascending pathways but in the feedback to V1 that was not appropriately masked. Admittedly, a stronger conclusion on this notion would require a direct study of the effect of the backward-masking on the presence of hemispheric asymmetry.

### Modeling as a New Diagnostic Tool

Our results challenge the “common sense” beliefs, e.g., that the extent of the VFD may predict the challenge posed to people with OCL, as Figure 5 shows that patient P07 with the largest VFD performs better than patient P01 with significant field sparing. The occluded part of the visual field in a healthy observer causes a significant drop in scene discrimination, which depends lawfully on the extent of the occlusion in healthy observers as well as in the GIST model. For OCL participants, this relationship is not so clear. The experience of a patient with a rather complete hemianopia can be casual: “The visual field defect, although annoying, does not really bother me. You cannot see behind you either, right?,” while others experience their (smaller) defect as a serious handicap and need to resort to a white cane for assistance. People with OCL do not experience VFD as a simple occlusion of the functional visual field. Rather peoples’ experiences range from not being able to give a meaningful response to stimuli in their VFD to being able to unconsciously process stimulus information, as in blindsight (Poppel et al., 1973; Weiskrantz et al., 1974). Clearly, a direct comparison like the extent of the VFD between OCL participants and healthy observers, leaves unexplained much of the ADL variation between patients. Further investigations may benefit from the use of a more elaborate approach, using more sophisticated stimulation combined with modeling.

Our method could distinguish three categories of OCL participants: category I includes about half of the OCL participants who perform significantly worse than the GIST model would predict based on the extent and size of their VFDs; category II, the other half of OCL participants, performed as predicted; and category III with one participant P07 who performed even better than the model predicts. When considering the GIST model, we appear to find a correlation in the anatomical location of their lesions. For category I, lesions include the posterior part of the corpus callosum (splenium) and subcortical areas including the LGN in addition to OCLs. In OCL participants from category II, the lesions are limited to the striate and extrastriate occipital areas. For category III, or rather the case study of participant P07, we observed a focal optic radiation lesion following a medical procedure to remove a knot of blood vessels. This lesion leaves (extra-)striate areas and the subcortical areas largely intact. This particular participant is also young (25 years old), and we observed that despite his rather complete hemianopia, he performed even better than the average healthy observer would when this participant’s VFD was simulated. We suggest that even though this patient cannot consciously process information in the field defect, the intact cortical tissue lacking input might still somehow contribute to global visual processing of the scene through, for example, interhemispheric communication, or through subcortical projections bypassing area V1 and directly projecting to extrastriate cortex.

The characteristics of OCL are usually determined with perimetry, which involves detection of flashed point stimuli. Such visual stimuli do not probe the spatial integration properties of the visual system and may underestimate the visual effect of damage to those properties. Therefore, we investigated performance on a visual scene discrimination task in OCL participants, which does require spatial integration. A functional model of scene discrimination (Torralba and Oliva, 2003) turned out to also provide an accurate description of the performance by healthy subjects with *simulations* of OCL by occlusion of a part of their visual field even when moderate levels of noise were applied. This allowed prediction of performance loss in real OCL participants on the basis of extent and location of their VFD and the GIST model. Note that this implies the assumption that the OCL visual network performs as in healthy subjects. We found in 17 OCL participants eight with performance loss as predicted by the GIST model, eight with significantly larger performance loss, and one subject with significantly better performance than expected from occlusion only.

## Conclusion

Our modeling approach provides a quantitative way to characterize OCL participants’ defects in a more naturalistic context than the standard visual field measurements. This is something that has been lacking in the present literature and in the clinic alike. We noticed that contrary to standard “common sense” beliefs, the extent or location of the VFD does not predict the challenge posed with respect to scene perception to the OCL participants. By using a modeling approach, we cautiously suggest that lesions affecting interhemispheric integration may contribute to the performance loss. We propose that the role of interhemispheric connections for dealing with people with OCL needs more attention.

## Data Availability Statement

The raw data supporting the conclusions of this article will be made available by the authors, without undue reservation.

## Ethics Statement

The studies involving human participants were reviewed and approved by Commissie Mensgebonden Onderzoek Regio Arnhem-Nijmegen (2016–2635). The patients/participants provided their written informed consent to participate in this study.

## Author Contributions

AG, KW, and AB contributed to conception and design of the study. AG developed the methods, wrote the software, and analyzed the data. AG and KW collected the data. AG and AB wrote the manuscript. All authors contributed to manuscript revision, read, and approved the submitted version.

## Conflict of Interest

The authors declare that the research was conducted in the absence of any commercial or financial relationships that could be construed as a potential conflict of interest.

## Publisher’s Note

All claims expressed in this article are solely those of the authors and do not necessarily represent those of their affiliated organizations, or those of the publisher, the editors and the reviewers. Any product that may be evaluated in this article, or claim that may be made by its manufacturer, is not guaranteed or endorsed by the publisher.

## Footnotes

1
http://people.csail.mit.edu/torralba/code/spatialenvelope/

## Abbreviations

V1: primary visual cortex
VFD: visual field defect
LGN: lateral geniculate nucleus
OCL: occipital cortex lesion.
