# Supplementary material for: When You Do Not Get the Whole Picture: Scene Perception After Occipital Cortex Lesions
Source: Front Neurosci. 2021 Dec 13;15:716273. doi: 10.3389/fnins.2021.716273 (PMC8710569; doi:10.3389/fnins.2021.716273)
Supplement: Supplementary file 1 [file Table_1.DOCX]

Supplementary Material

**Table S1. Humphrey’s test reliability indexes**. Percentage False Positives, False Negatives, and fixation losses for the left and right eye individually. ^*^Low test reliability of right eye. For these participants only the left Humphrey perimetry has been used. ^**^Low test reliability for both eyes, for lack of a better solution we use the available measurement.

| **OCL no.** | **False positives** | | **False**  **negatives** | | **Fixation**  **loss** | |
| --- | --- | --- | --- | --- | --- | --- |
|  | **L** | **R** | **L** | **R** | **L** | **R** |
| P01 | 0 | 1 | 12 | 0 | 8 | 13 |
| P02 | 0 | 1 | 0 | 16 | 0 | 8 |
| P03 | 0 | 1 | 0 | 0 | 0 | 14 |
| P04 | 1 | 2 | 0 | 12 | 0 | 0 |
| ***P05^**^*** | ***9*** | ***15*** | ***20*** | ***27*** | ***93*** | ***43*** |
| P06 | 1 | 0 | 0 | 0 | 0 | 15 |
| P07 | 0 | 0 | 0 | 0 | 0 | 0 |
| P08 | 1 | 3 | 0 | 0 | 0 | 12 |
| P09 | 0 | 0 | 0 | 0 | 6 | 0 |
| P10 | 5 | 0 | 0 | 0 | 0 | 17 |
| P11 | 0 | 0 | 0 | 0 | 0 | 0 |
| P12 | 0 | 0 | 0 | 0 | 0 | 7 |
| P13 | 0 | 1 | 0 | 0 | 0 | 0 |
| P14 | 0 | 3 | 0 | 0 | 7 | 0 |
| P15 | 0 | 1 | 0 | 0 | 0 | 0 |
| **P16^*^** | 0 | 2 | 0 | 0 | 0 | ***71**** |
| **P17^*^** | 2 | 0 | 0 | 0 | 0 | ***65**** |

|  | **Lesion description** |
| --- | --- |
| **P01:** | Both striate, extrastriate areas and a large part of the optic radiation are damaged. It also shows damage of the hippocampus, LGN and the corpus callosum. |
| **P02:** | - |
| **P03:** | - |
| **P04:** | - |
| **P05:** | A complex lesion after a bilateral trephination of the partial and occipital cortex and an increased size of ventricles. The striate (V1) and extrastriate occipital areas (V2-V4) are intact, but the corpus callosum and overall, the larger part of subcortical nuclei including the LGN are damaged. |
| **P06:** | Damage of V1 and V2, very small, potential damage on horn of the corpus callosum. |
| **P07:** | No scan present, but patient has a focal lesion of optic radiation because of the removal of a knot of blood vessels. |
| **P08:** | Lesion affects V1/V2, the optic radiation and is a rather deep defect extending up to corpus callosum. |
| **P09:** | Damage of V1 and a small part of V2. |
| **P10:** | CT-scan does not reveal any damage, but the spatial resolution is not sufficient to draw proper conclusions. |
| **P11:** | Damage of V2/V3 and optic radiation, V1, LGN and corpus callosum are intact |
| **P12:** | Damage of V1/V2 and the optic radiation, but subcortical areas including LGN and the corpus callosum are intact. |
| **P13:** | V1/V2, a small part of V3 and the optic radiation are damages. The subcortical areas are intact, but the lesion shows to include a small part of the corpus callosum. |
| **P14:** | Damage of V1, a small part of V2 and the optic radiation. Subcortical areas are intact. |
| **P15:** | Incomplete structural scan, but V1/V2 are clearly damaged. The image shows a rather deep defect extending into the subcortical areas, possibly affecting the corpus callosum. |
| **P16:** | A focal lesion of the LGN and beginning of the optic radiation. |
| **P17:** |  |

**Table S2. Description of the extent and location of the lesion in the structural scans**.

**

**

**Figure S1 Structural scans** of 12 OCL participants, showing the extent and size of the lesions in red sorted by performance on the scene perception task for worst to best. Note that P10 CT-scan does not reveal any damage.
